# Effect of collagen vascular disease-associated interstitial lung disease on the outcomes of lung cancer surgery

**DOI:** 10.1007/s00595-017-1476-z

**Published:** 2017-02-28

**Authors:** Hideyuki Maeda, Masato Kanzaki, Kei Sakamoto, Tamami Isaka, Kunihiro Oyama, Masahide Murasugi, Takamasa Onuki

**Affiliations:** Department of Surgery I, Tokyo Women’s Medical University, 8-1 Kawada-cho, Shinjuku-ku, Tokyo, 162-8666 Japan

**Keywords:** Lung cancer, Surgery, Interstitial lung disease, Collagen vascular disease

## Abstract

**Purpose:**

This study compared the effect of collagen vascular disease-associated interstitial lung disease (CVD-ILD) with that of idiopathic interstitial pneumonias (IIPs) on the outcomes of lung cancer surgery.

**Methods:**

This study retrospectively reviewed the medical records of patients who underwent surgery for non-small cell lung cancer (NSCLC) and compared the data of 16 patients with CVD-ILD with those of 70 patients with IIPs. The patterns of interstitial lung disease (ILD) on chest computed tomography were classified into usual interstitial pneumonia (UIP) and non-specific interstitial pneumonia (NSIP) patterns.

**Results:**

The numbers of UIP and NSIP patterns were 10 (62.5%) and 6 (37.5%) patients in CVD-ILD group, and 62 (88.6%) and 8 (11.4%) patients in IIPs group, respectively. A postoperative acute exacerbation (AE) appeared in 1 patient (6.3%) in the CVD-ILD group and 6 patients (8.6%) in the IIPs group. No significant differences in the incidence of postoperative AE and mortalities were observed between the two groups. The five-year overall survival rates of the CVD-ILD and IIPs groups were 37.5 and 49.2%, respectively.

**Conclusions:**

Surgery for NSCLC in CVD-ILD patients appear to cause no increase in postoperative AE and mortality in comparison to that seen in IIPs patients. Similar to IIPs, CVD-ILD might therefore affect the prognosis of resected NSCLC.

## Introduction

Interstitial lung disease (ILD) frequently coexists with collagen vascular disease (CVD) in patients, and most such patients are treated with immunosuppressive agents. Lung cancer in patients with ILD has a poor prognosis, and the postoperative acute exacerbation (AE) of ILD is one of the most critical complications observed after lung resection. Recently, Fukui et al. report that patients with combined pulmonary fibrosis and emphysema show poor postoperative outcomes after lung cancer surgery [1]. Although the prognosis of CVD-associated ILD (CVD-ILD) is better than that of idiopathic interstitial pneumonias (IIPs) [2], the effect of CVD-ILD on the prognosis of lung cancer compared to that of IIPs and the incidence of postoperative AE of CVD-ILD are unknown. In addition, the use of immunosuppressive agents may increase the risk of postoperative complications, such as bronchopleural fistula (BPF). Consequently, CVD-ILD patients undergoing lung cancer surgery are speculated to have a higher risk of postoperative morbidities and mortality. However, to date, there is no report regarding the outcomes of lung cancer surgery in CVD-ILD patients. This study compared the effect of CVD-ILD with that of patients with IIPs on the outcomes of lung cancer surgery.

## Patients and methods

**Fig. 1:**
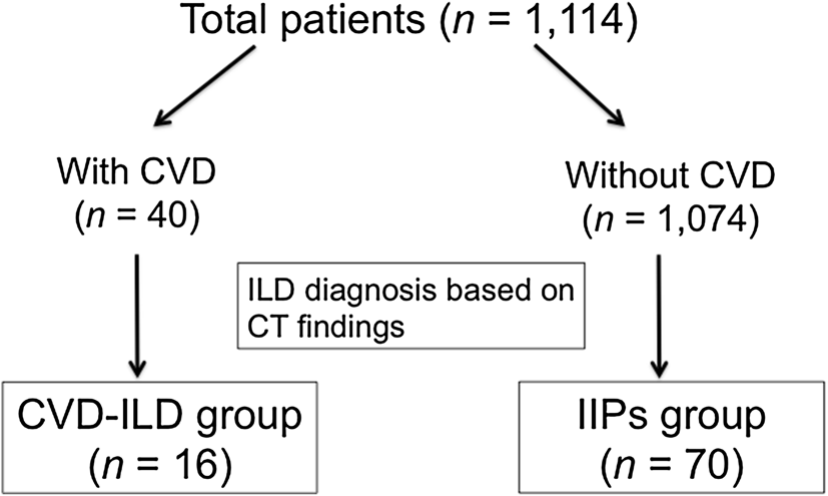
Study enrolment flow chart. CT indicates computed tomography; CVD, collagen vascular disease; CVD-ILD, collagen vascular disease associated interstitial lung disease; IIPs, idiopathic interstitial pneumonias; ILD, interstitial lung disease

This was a single-institutional retrospective study based on medical records obtained from the authors’ institution. The authors reviewed the clinical data of 1114 patients who underwent lung resection for non-small cell lung cancer (NSCLC) at our institution from January 2003 to December 2014. These patients were classified into two groups, including patients with CVD (*n* = 40) and those without (*n* = 1074). Then, based on the chest computed tomography (CT) findings described below, the patients belonging to the CVD-ILD group (*n* = 16) and those belonging to the IIPs group (*n* = 70) were newly extracted from the two groups individually (Fig. 1). The postoperative follow-up period was defined as the period from operation to the last follow-up appointment.

### Diagnosis of interstitial lung disease

The diagnosis of ILD was based on the following criteria, and the patterns of ILD on chest CT was classified into usual interstitial pneumonia (UIP) and non-specific interstitial pneumonia (NSIP) patterns. The criteria for the UIP pattern were (1) subpleural basal predominance, (2) reticular abnormality, (3) honeycombing with or without traction bronchiectasis, and (4) the absence of features listed as inconsistent with UIP pattern as described in the literature [3]. Any patients with a possible UIP pattern on CT [3] were excluded from this study. The criteria for the NSIP pattern were reticular opacities with a lower lung zone predominance, associated with traction bronchiectasis, and lobar volume loss [4]. CT scanned images were read by both experienced radiologists and thoracic surgeons. After surgery, all resected specimens were investigated by pathologists to confirm the existence of ILD.

### Prophylaxis for an acute exacerbation of interstitial pneumonia

At the authors’ institution, ulinastatin, as a prophylactic treatment for postoperative AE after pulmonary resection, was used for patients with simultaneous lung cancer and ILD [5]. Two days before surgery, ulinastatin was administered as a 30-min infusion of 300,000 units (U) for three consecutive days. The dose of ulinastatin for days 4 to 6 was reduced to 200,000 U, and that for days 7 –9 was further reduced to 100,000 U.

### Definition of postoperative event

Postoperative complications were defined as complications that appeared within 30 days after surgery and included (1) arrhythmia requiring intervention, and (2) prolonged air leakage persisting for 7 days postoperatively and requiring pleurodesis. In addition, the 30- and 90-day mortalities were defined as death within 30 and 90 days after surgery, respectively, and included any instances of death after discharge.

Postoperative AE was diagnosed according to the following diagnostic criteria: (1) an unexplained worsening or the development of dyspnea within 30 days, (2) a new bilateral ground-glass abnormality and/or consolidation superimposed on a background reticular or honeycomb pattern on CT scan, (3) worsening hypoxemia from a known baseline arterial blood gas level, (4) the absence of infectious disease, (5) the exclusion of alternative causes including left heart failure and pulmonary embolism and (6) the identifiable cause of acute lung injury [3, 6]. Patients with postoperative AE received steroid pulse therapy with methylprednisolone as the first line treatment.

### Statistical analysis

Categorical data were presented as numbers and percentages, and continuous data were presented as the median and interquartile range (IQR). For comparing CVD-ILD and IIPs groups, the categorical data were analyzed by the Fisher’s exact test or the Chi-square test, and continuous data were analyzed by the Mann–Whitney *U* test. Overall survival (OS) was defined as the time from the operation to death from any cause, and the 5-year OS rate was estimated by the Kaplan–Meier method. Differences between the two groups were analyzed by the log-rank test. Probabilities of less than 0.05 (*p* < 0.05) were considered statistically significant. All statistical analyses were performed by JMP Pro 12.1 statistical software (SAS Institute, Cary, NC, USA).

### Ethics

This study was approved by the Research Ethics Committee of the Tokyo Women’s Medical University, Tokyo, Japan (No.3852).

## Results

Sixteen patients with CVD-ILD underwent lung resection for NSCLC at the authors’ institution during the study period and their baseline characteristics are given in Table 1. The median age was 66.5 years (IQR: 16 years), 6 patients (37.5%) were men, and 10 patients (62.5%) were women. There were significant differences in gender (*p* = 0.0201) and the Brinkman index (*p* = 0.0057) between the CVD-ILD and IIPs groups. ILD patterns on chest CT in CVD-ILD group were found in 10 patients with a UIP pattern, and in 6 patients with an NSIP pattern. No patients received induction chemotherapy. Pathological examinations of resected specimens showed that 7 UIP pattern cases with rheumatoid arthritis (RA) were found in the CVD-ILD group, 18 UIP pattern cases were found in the IIPs group, and one NSIP pattern was found. The median follow-up period was 34.0 months (IQR: 42.8 months). There was no significant difference in the ILD pattern on CT between the two groups. In the CVD-ILD group, RA was the most common CVD (Table 2). Of those, 13 patients (81.3%) received immunosuppressive therapy consisting of prednisolone (PSL), methotrexate (MTX), tacrolimus (FK506), and mizoribine (MZ). In the immunosuppressant regimens, PSL (*n* = 8) and MTX (*n* = 2) were used as a monotherapy, and PSL with FK506 (*n* = 1), PSL with MZ, and MTX with MZ were used individually as a combination therapy.

**Table 1:** Comparison of the patient characteristics

Variables	CVD-ILD group	IIPs group	*p* value*
Total number of patients	16	70	
Age (years)	66.5 (16)**	73.0 (11.3)**	0.132
Gender			0.0201
Male	6 (37.5%)	62 (88.6%)	
Female	10 (62.5%)	8 (11.4%)	
Brinkman index	600 (900)**	1000 (1000)**	0.0057
Number of comorbidities			0.119
1	0	8 (11.4%)	
2	10 (62.5%)	31 (44.3%)	
3	6 (37.5%)	19 (27.2%)	
4≤	0	12 (17.1%)	
ILD pattern on CT			0.0709
UIP pattern	10 (62.5%)	58 (82.9%)	
NSIP pattern	6 (37.5%)	12 (17.1%)	
%VC	109.7 (40.4)**	108.7 (34.5)**	0.893
%DLco	57.4 (16.1)**	55.5 (27.6)**	0.478
KL-6 (U/mL)	481 (342.5)**	514 (440)**	0.699
Neoadjuvant chemotherapy	0	2 (2.9)	0.494
Pathology			0.614
Adenocarcinoma	9 (56.3%)	30 (42.9%)	
Squamous cell carcinoma	5 (31.2%)	30 (42.9%)	
Others	2 (12.5%)	10 (14.2%)	
Pathological stage			0.153
I	9 (56.2%)	40 (57.1%)	
II	4 (25%)	11 (15.7%)	
III	2 (12.5%)	19 (27.2%)	
IV	1 (6.3%)	0	
Median follow-up (months)	34 (42.8)**	30.5 (35.5)**	0.89

*p values were calculated by Fisher’s exact test or the Chi-square test for categorical data, or Mann–Whitney’s *U* test for continuous data

**Continuous variables are expressed as the median and interquartile ranges which are expressed in parenthesis, and categorical variables are expressed as numbers and percentages (%). CVD-ILD indicates collagen vascular disease-associated interstitial lung disease

*IIPs* idiopathic interstitial pneumonias, *ILD* interstitial lung disease, *CT* computed tomography, *UIP* usual interstitial pneumonia, *NSIP* non-specific interstitial pneumonia, *VC* vital capacity, *DL*_*CO*_ carbon monoxide diffusion capacity, *KL-6* Krebs von den Lungen-6

**Table 2:** Types of collagen disease and the distribution of ILD patterns

Collagen Disease	ILD pattern on CT		The number of cases
	UIP pattern	NSIP pattern	
Rheumatoid arthritis (RA)	9	4	13
Sjögren syndrome (SjS)	1	0	1
Dermatomyositis (DM)	0	1	1
Systemic lupus erythematosus (SLE)	0	1	1

*ILD* interstitial lung disease, *CT* computed tomography, *UIP* usual interstitial pneumonia, *NSIP* non-specific interstitial pneumonia

The surgical and clinical outcomes are shown in Table 3. The most common operative procedure was lobectomy (*n* = 8), followed by segmentectomy (*n* = 5), and wedge resection (*n* = 3) in the CVD-ILD group. R0 resection was achieved in all patients except for one patient with pathological stage IV disease in the CVD-ILD group. The reinforcement of the bronchial stump in major lung resection was performed in 4 of 8 cases (50%) in the CVD-ILD group and in 2 of 44 cases (4.5%) in IIPs group (*p* = 0.0002), and a pedicled intercostal muscle flap was used in all cases. In the CVD-ILD group, no intraoperative complications appeared, while postoperative complications were observed in 4 patients. Postoperative AE developed in 1 patient (6.3%), and BPF developed in 2 patients (12.5%). Patients with BPF underwent the reinforcement of the bronchial stump because of the use of immunosuppressive agents. The thirty- and ninety-day mortalities were 6.3%, because one patient died with postoperative AE (Table 3). Regarding the postoperative complications, no significant difference in the incidence of postoperative AE and a significant difference in the incidence of BPF (*p* = 0.0028) were observed between both groups. No significant differences in hospital stay, the incidence of postoperative complications, and 30- and 90-day mortalities were found between either of the groups (Table 3).

**Table 3:** Comparison of the surgical and clinical outcomes

Variables	CVD-ILD group (*n* = 16)	IIPs group (*n* = 70)	*p* value*
Operative approach			0.766
VATS	12 (75%)	47 (67.1%)	
Thoracotomy	4 (25%)	23 (32.9%)	
Operative method			0.418
Wedge resection	3 (18.7%)	18 (25.7%)	
Segmentectomy	5 (31.3%)	8 (11.5%)	
Lobectomy	8 (50%)	40 (57.1%)	
Bilobectomy	0	3 (4.3%)	
Pneumonectomy	0	1 (1.4%)	
Operative time (min)	163.5 (115.8)**	189 (117.5)**	0.682
Loss (mL)	92.5 (340.5)**	145 (281)**	0.915
Intraoperative complication	0	2 (2.9%)	0.494
Duration of drainage (days)	4 (3)**	4 (3)**	0.0777
Length of stay (days)	11 (10.5)**	12 (10)**	0.96
Postoperative complication			
Overall	4 (25%)	19 (27.1%)	0.861
AE of ILD	1 (6.3%)	6 (8.6%)	0.759
Prolonged air leakage	3 (18.8%)	6 (8.6%)	0.23
Pneumonia	0	3 (4.3%)	0.399
BPF	2 (12.5%)	0	0.0028
Reintubation	1 (6.3%)	2 (2.9%)	0.505
Tracheostomy	1 (6.3%)	2 (2.9%)	0.505
Arrhythmia	1 (6.3%)	1 (1.4%)	0.248
Others	0	4 (5.7%)	0.328
30-day mortality	1 (6.3%)	2 (2.9%)	0.505
90-day mortality	1 (6.3%)	4 (5.7%)	0.934

*p values were calculated by Fisher’s exact test or the Chi-square test for categorical data, or Mann–Whitney’s *U* test for continuous data

**Continuous variables are expressed as the median and interquartile ranges which are expressed in parenthesis, and the categorical variables are expressed as numbers and percentages (%)

*CVD-ILD* indicates collagen vascular disease-associated interstitial lung disease, *IIPs* idiopathic interstitial pneumonias, *VATS* video-assisted thoracoscopic surgery, *AE* acute exacerbation, *ILD* interstitial lung disease, *BPF* bronchopleural fistula

**Fig. 2:**
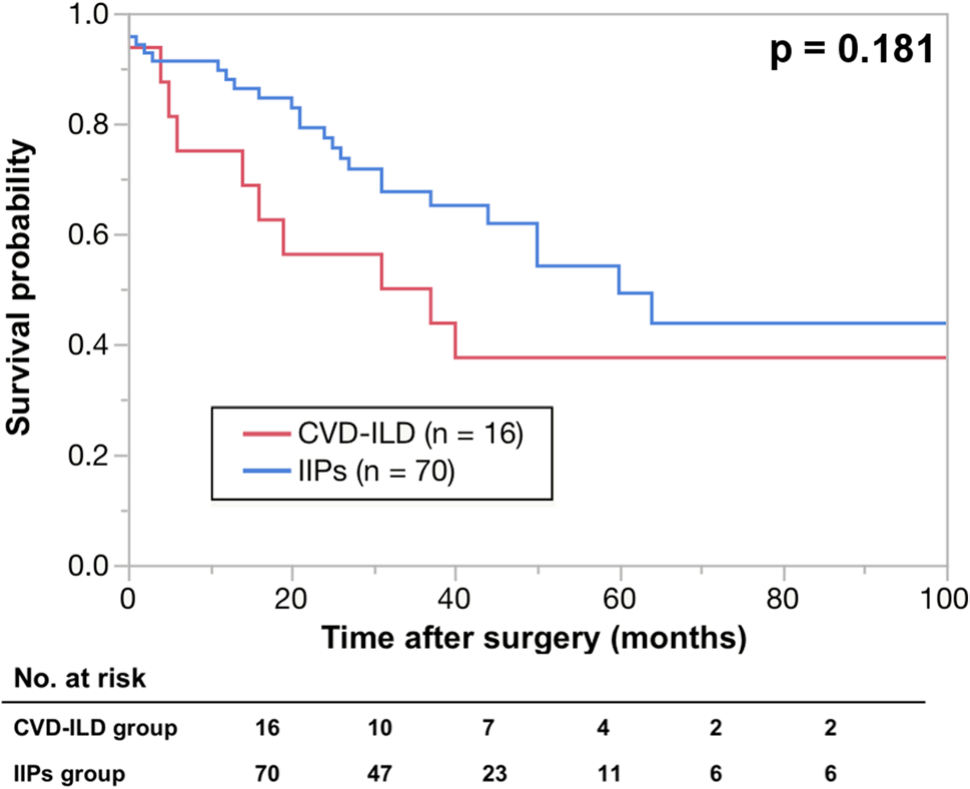
Kaplan-Meier survival curves of collagen vascular disease-associated interstitial lung disease (CVD-ILD) and idiopathic interstitial pneumonias (IIPs) groups. The 5-year overall survival rates in patients with interstitial lung disease were 35.2% in CVD-ILD group (n = 16) (red line) and 45.1% in IIPs group (n = 70) (blue line)

**Fig. 3:**
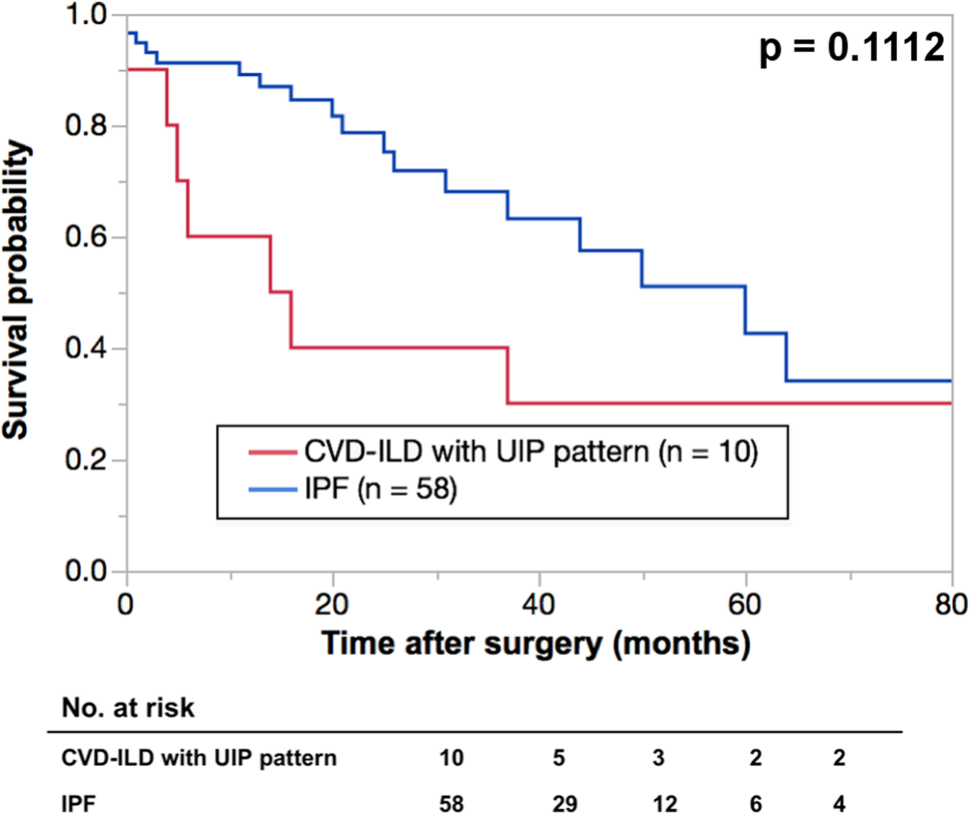
Kaplan-Meier survival curves of collagen vascular disease-associated interstitial lung disease (CVD-ILD) with usual interstitial pneumonia (UIP) pattern and idiopathic pulmonary fibrosis (IPF) groups. The 5-year overall survival rates in patients with interstitial lung disease were 30% in CVD-ILD patients with UIP pattern (n = 10) (red line) and 42.5% in IPF group (n = 58) (blue line)

**Fig. 4:**
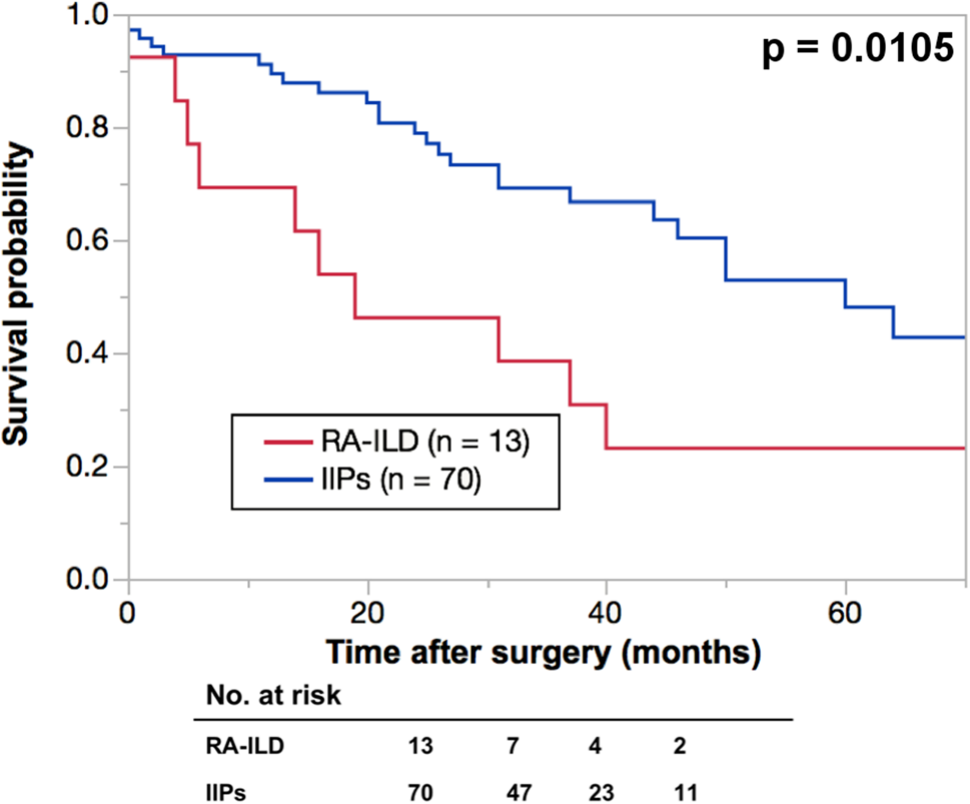
Kaplan-Meier survival curves of rheumatoid arthritis-associated interstitial lung disease (RA-ILD) and idiopathic interstitial pneumonias (IIPs) groups. The 5-year overall survival rates in patients with interstitial lung disease were 23% in RA-ILD (n = 10) (red line) and 49.2% in IIPs group (n = 70) (blue line)

The five-year OS rates were 37.5% in the CVD-ILD group and 49.2% in the IIPs group (Fig. 2), and there was no significant deference between the two groups. A total 10 deaths after surgery were observed in the CVD-ILD group during the observation period, and the causes in the CVD-ILD group were compared with those in the IIPs group (Table 4), and ILD was the second common cause of death after lung cancer in both groups. This study also compared the OS rates between CVD-ILD with a UIP pattern and IPF patients, and between RA-associated ILD (RA-ILD) and IIPs patients (Figs. 3, 4). The five-year OS rates were 30% in the CVD-ILD patients with a UIP pattern and 42.5% in the IPF patients, and no significant difference was found between them (Fig. 3). The five-year OS rates were 23% in the RA-ILD patients and 49.2% in the IIPs patients, and there was a significant difference between the two groups (*p* = 0.0105) (Fig. 4).

**Table 4:** Details of the cause of death

Cause	CVD-ILD group (*n* = 10)	IIPs group (*n* = 26)	*p* value
			0.769
Lung cancer (%)	5 (50)	16 (61.5)	
ILD (%)	3 (30)	7 (27)	
Postoperative AE	1	6	
AE occurring ≥POD 31	2	1	
Others (%)	2 (20)	3 (11.5)	

*CVD-ILD* indicates collagen vascular disease-associated interstitial lung disease, *IIPs* idiopathic interstitial pneumonias, *ILD* interstitial lung disease, *AE* acute exacerbation, *POD* postoperative day

## Discussion

To date, the clinical features and prognosis of CVD-ILD with those of IIPs are studied and reported by several investigators. Among them, Park et al. report that (1) the common types of CVD-ILD are a UIP and non-specific interstitial pneumonia pattern according to the classification of IIPs and (2) a UIP pattern is found to more frequently coexist with RA than other CVD types [7]. Park et al. also report the (1) 3- and 5-year survival rates to be better in patients with a UIP pattern in the CVD group than those of the IIPs group, but (2) no significant difference was found between the patients with an NSIP pattern in both groups [2]. The report also suggests that IIPs may affect the prognosis of lung cancer patients more strongly than CVD-ILD. However, this study obtained the opposite results. Although no significant difference in the 5-year OS rates between CVD-ILD patients and IIPs patients was found (Fig. 2), the CVD-ILD group showed a lower OS rate. Further analysis found that (1) CVD-ILD patients with a UIP pattern showed a lower OS rate than IPF patients (Fig. 3) and (2) RA-ILD patients had a significantly lower OS rate than IIPs patients (Fig. 4), indicating that IIPs failed to affect the prognosis of lung cancer patients more strongly than CVD-ILD. For explaining the lower OSs in patients with CVD-ILD in this study, the following reasons were speculated. First, the state of ILDs or CVD was ignored in the survival analysis, and this might have caused some bias in selecting the adjuvant chemotherapy and the treatment of recurrence of lung cancer. Second, as Sumikawa et al. [8] and Sverzellati [9] reported, there could be a discrepancy between the diagnosis of ILDs based on the CT and pathological findings. Even in this study, CVD-ILD group included 7 patients with a UIP pattern, and the IIPs group included 18 patients with a UIP pattern. Therefore, some patients showing a UIP pattern on CT might demonstrate a UIP pattern in the pathological findings. This inconsistency might affect OS. Since the resected specimens did not always include the area of ILD, the pathological diagnoses of ILDs were thus considered to be difficult to confirm in all cases. Therefore, physicians must realize the importance of multidisciplinary discussions with pathologists in diagnosing ILDs. Another important reason to explain why the lower OSs were found in patients with CVD-ILD in this study appeared to be due to a difference in the sample size.

There are three reports regarding the 5-year survival rates in lung cancer patients with IIPs, and the 5-year survival rates in patients with NSCLC and IPF, stage IA NSCLC and IPF, and IPF and stage IA and IB NSCLC were 52% [10], 54.2% [11], and 61.6% [12], respectively. A multicenter retrospective study with a large numbers of patients with ILD who underwent lung cancer surgery shows that the 5-year overall survival rates are 40.0% [13]. As described above, even though the diagnosis of ILD based on the CT findings might influence the results, the 5-year survival rates in the lung cancer patients with IIPs in this study showed no major difference from the reported results, and at least, similar to IIPs, this study showed that CVD-ILD might affect the long-term results of lung cancer surgery.

Lung resection in CVD-ILD patients could lead to postoperative AE, which is a fatal complication. However, these patients may be unable to obtain as much of a benefit as that provided by the other treatment modalities including chemotherapy and radiation therapy. Kenmotsu et al. [14] report that 9% of 104 NSCLC patients with ILD who are treated with platinum-based chemotherapy develop a chemotherapy-related exacerbation during the first-line chemotherapy. Although molecular targeting drugs, such as epidermal growth factor receptor tyrosine kinase inhibitor and anaplastic lymphoma kinase inhibitor, have recently been used to treat NSCLC, these agents can cause an exacerbation of ILD as an adverse effect. In addition, a dose reduction may be considered, because bone marrow suppression after chemotherapy in CVD-ILD patients who received immunosuppressive agents can lead to the development of severe infectious diseases. Ozawa et al. [15] report that pre-existing interstitial disease is a predictive factor of extensive radiation pneumonia. From these results, chemotherapy and radiation therapy for lung cancer with CVD-ILD are therefore not considered to be a more effective treatment modality than surgery. Therefore, this study considered that lung cancer patients with CVD-ILD would be candidates for surgical treatment.

The prediction and prevention of postoperative AE is quite important. Several reports suggest that the following items could be predictors of postoperative AE; patient age, the duration of postoperative drainage and intraoperative fluid balance [16–18], typical honeycombing and possible honeycombing patterns on the chest CT findings [19], and the I/M ratio, which is the ratio of the peak standardized uptake value (SUV) of the interstitial lung disease area to the mean SUV of the mediastinum [20]. Although medications are used to prevent postoperative AE of ILD [5, 21], no evidence for prophylaxis has yet been established. Ulinastatin, as a prophylactic agent used in this study for postoperative AE, showed the incidence rates of postoperative AE in this study to be 6.3% in the CVD-ILD group and 2.9% in the IIP group, and these results were more favorable than the 9.3% rate reported in a multicenter study in Japan reported by Sato et al. [22]. Recently, the effect of perioperative pirfenidone treatment for preventing postoperative AE of ILD has been reported [23–25], further investigations are expected. Hozumi et al. [26] investigate the risk factor of AE of ILD in RA patients, and a univariate analysis revealed that an older age at the time of interstitial pneumonia diagnosis, the UIP pattern on HRCT, and MTX usage are associated with AE of ILD. This study was unable to identify any predictors of AE of ILD in patients with CVD-ILD, because postoperative AE appeared in only one patient with CVD-ILD. However, Sato et al. [22] report that preoperative steroid use is an independent risk factor of postoperative AE, and in this study, the use of immunosuppressive agents in CVD-ILD patients might be a risk factor of postoperative AE. Describing the severity of postoperative AE of ILD is also important, but it is difficult because there is no report about grading postoperative AE. Katayama et al. reported on the Japan Clinical Oncology Group postoperative complications criteria, including six thoracic complications [27]. Postoperative AE is not included in these criteria, however, grading postoperative AE may be a useful tool when thoracic surgeons make an investigation concerning postoperative AE. Future considerations should thus be expected.

Besides being an essential for treating CVD, immunosuppressive agents can cause some problems in the perioperative and long-term periods for CVD patients undergoing lung resection. First, infectious complications such as pneumonia caused by immunosuppression, prolonged air leakage, and BPF due to the inhibition of wound healing could be induced by immunosuppressive agents. Prolonged air leakage can cause an infection in the intrapleural space, leading to pyothorax. The temporary discontinuation of immunosuppressive agents could be acceptable as long as the state of CVDs is stable, and consultations with rheumatologists are therefore important before surgery. In this study, the CVD-ILD group showed a higher incidence of BPF than the IIPs group in spite of the reinforcement of the bronchial stump, this higher incident rate would be a risk factor for inducing serious problems, because BPF can lead to fatal pyothorax and broncho-pulmonary artery fistula (BPAF). Pyothorax developed in CVD-ILD patients treated with immunosuppressive agents and it was expected to become exacerbated. Although bronchial closure methods and a predictive score for BPF have been reported [28, 29], no evidence showing the effectiveness of the reinforcement of the bronchial stump to prevent BPF is available. In fact, two patients who developed BPF in the CVD-ILD group were applied with the reinforcement of the bronchial stump. In the authors’ department, this procedure was routinely performed for high-risk patients including patients suffering from severe diabetes mellitus, took immunosuppressive agents, or received neoadjuvant chemoradiation therapy, because it was considered possible to avoid fatal BPAF by isolating the bronchial stump and pulmonary artery. Second, calcineurin inhibitors, such as tacrolimus could worsen the prognosis of cancer over the long term, because these agents induce tumor growth, invasion, and metastasis [30]. In this study, because only one patient took tacrolimus, a calcineurin inhibitor, this study was unable to obtain any conclusive results regarding these inhibitors, because the number of observations was insufficient. Therefore, future investigations are called to clarify their effect. Third, the doses of immunosuppressive agents can be increased upon the exacerbation of the CVD status, and bone marrow suppression due to chemotherapy may lead to a critical infection. Therefore, a decrease in the dose of chemotherapy results in an insufficient curative effect, thereby affecting the prognosis.

Because this study was a retrospective single institutional study, the number of cases investigated might be insufficient, and the small sample size could be a limitation associated with this study. The results of this study should be confirmed in a multicenter clinical study with a larger number of patients. The diagnosis of ILD in this study was based on the CT findings, because the resected specimens did not always include the area of ILD. There were a variety of CVD types and immunosuppressive treatment regimens in this study, and further investigations with a sufficient number of cases are called in the future.

## Conclusion

In conclusion, this study speculated that the incidence rate of postoperative AE of ILD and mortality in CVD-ILD patients after lung cancer surgery were not higher than those of IIPs patients. CVD-ILD patients might thus have poor prognosis after lung resection for NSCLC similar to that seen in IIPs patients.
